# Case Report: DARS Mutations Responsible for Hypomyelination With Brain Stem and Spinal Cord Involvement and Leg Spasticity

**DOI:** 10.3389/fgene.2022.845967

**Published:** 2022-04-26

**Authors:** Meijun Liu, Wen Xiao, Fang Yang, Xueqing Wang, Chao Chen, Shuoguo Jin, Ningjing Ran, Weiyin Chen, Dongdong Yang

**Affiliations:** Neurology Department, Hospital of Chengdu University of Traditional Chinese Medicine, Chengdu, China

**Keywords:** posterior leukoencephalopathy sgndrome, HBSL, DARS1, mutation, case reprot

## Abstract

**Objective:** Hypomyelination with brain stem and spinal cord involvement and leg spasticity (HBSL) is a rare form of leukodystrophy presenting with varying clinical and imaging features. We report a case of HBSL to investigate the clinical and radiological characteristics of HBSL resulting from cytoplasmic aspartyl-tRNA synthetase gene (DARS) mutations.

**Subjects:** We report a patient of HBSL with compound heterozygous mutations in DARS1. To study the potential genetic variations of the patient, targeted next-generation sequencing, whole-exome sequencing, and Sanger sequencing were used. We reviewed the clinical and radiological features of the patient. The literature was thoroughly evaluated.

**Results:** The patient suffered from developmental regression associated with lower limbs spasticity, developmental delay, and paralysis of the lower limbs since childhood. Decreased T1 and increased T2 signals were observed on the bilateral basal, centrum ovale, frontal lobe, parietal lobe, and ganglia in cervical cord magnetic resonance imaging (MRI). The patient had two compound heterozygous mutations (NM_001349:c.1363T > C and NM_001349:c.821C > G) in the DARS1 gene.

**Conclusion:** Two mutations in DARS1 were found to be associated with HBSL, one of them being reported for the first time. These findings can be valuable for diagnosing and providing genetic counseling to HBSL patients in the future.

## 1 Introduction

HBSL was first reported in 2013 (Ryan et al., 2013) in an autosomal recessive inheritance (Mendelian Inheritance in Man (OMIM): 6,15,281). Currently, HBSL is a rarely reported condition, and the occurrence of HBSL associated with the mutation in DARS is presented in a few studies (Ryan et al., 2013; Nicole et al., 2015). One of the involved enzymes is cytosolic aspartyl-tRNA synthetase DARS (DARS-AspRS), which pairs aspartate with its corresponding tRNA, and its expression is most pronounced in brain tissue (Ryan et al., 2013). Therefore, missense mutations in the gene encoding DARS result in the occurrence of HBSL with a distinct pattern of hypomyelination, motor abnormalities, and cognitive impairment (Zhang et al., 2018). In addition, MRI is characterized by broad symmetry high signal in T2Wimages and iso-intensity in T1W images of supratentorial white matter, brain stem, and cerebellar region. High signals in T2W images are observed in the dorsal spinal cord.

## 2 Patient and Result

**FIGURE 1:**
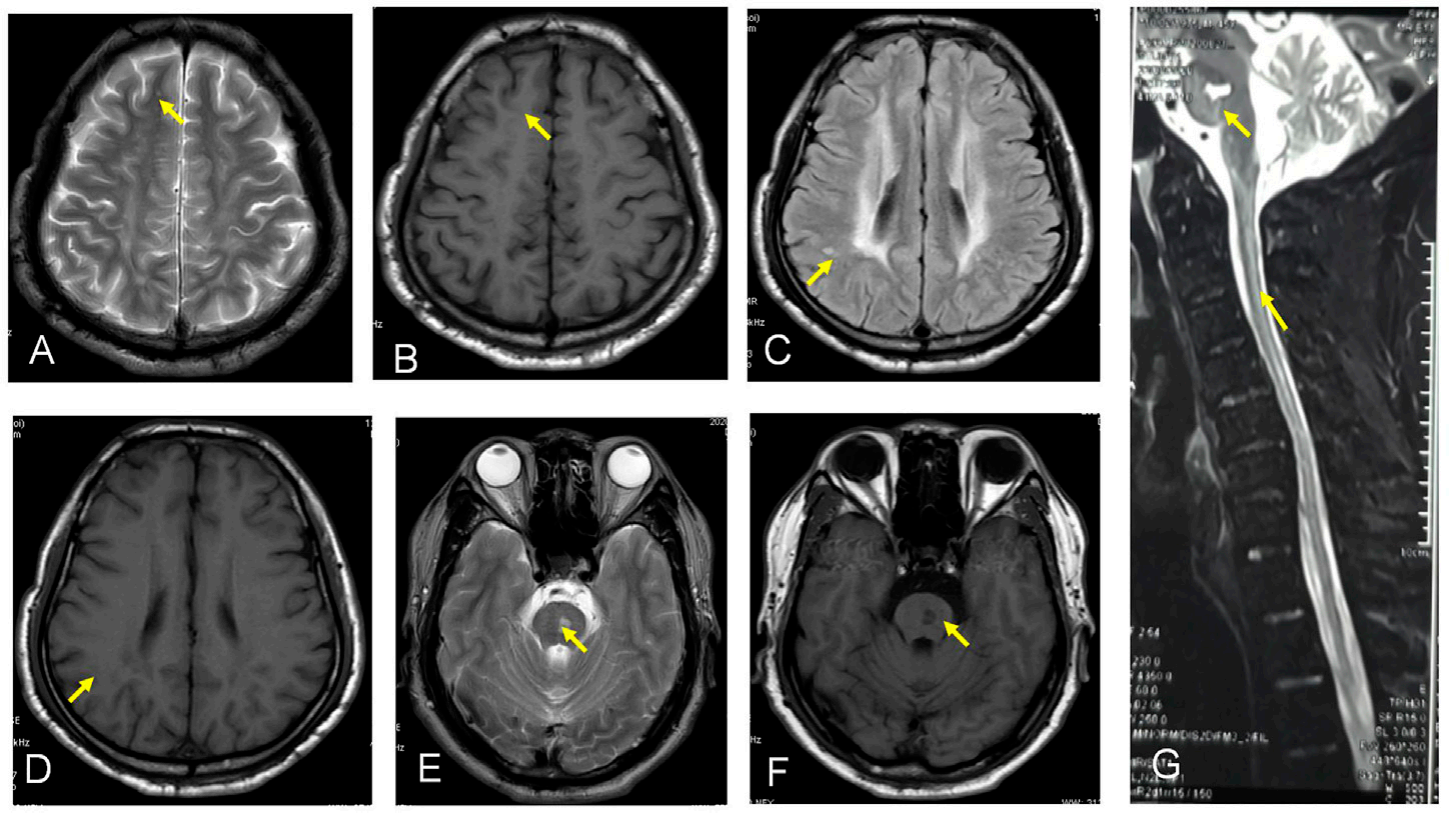
**(A–G)** show the MRI by a decreased signal on the T1 sequence and increased signal intensity on the T2/FLAIR sequance showed a of the bilateral basal, certain ovale, frontal lobe, and parietal lobe, and F shows increased signal on the T2 sequence showed a of ganglia cervical cord.

We report a case of HBSL in a 45-year old Han Chinese man of Sichuan Province. He had a history of normal birth and development of bilateral lower limb paralysis and delay in motor functions since 2 years of age. He complained of difficulty in walking, abnormal posture, and difficulty in standing after squatting down. By the age of 4 years, the patient could walk only with support, and by 5 years of age, he required support to stand as well. However, both the upper limbs had normal motor and cognitive functions. The patient did not receive any treatment due to poor economic background leading to a delay in diagnosis and treatment. He came for medical advice 1-year back due to progressive weakening in both upper limbs. At the time of consultation, the patient was unable to stand up, faced difficulty in raising upper limbs, and his cognitive function was deranged. On physical examination, findings were muscle strength grade 3 in both the upper limbs, hypermyotonia, hyperreflexia in bilateral upper and lower limbs (+++), positive bilateral Hoffman sign, higher muscle tension in both lower limbs, and a positive bilateral Babinski sign. Laboratory examination revealed plasma thrombin time 15.8 s, rheumatoid factor 21 IU/ml, and blood transferrin level 1.79 g/L. Routine blood test, urine test, and stool test were within normal limits. Liver and kidney function tests, electrolytes, tumor marker, and autoimmune antibody profile revealed no abnormality. An electrocardiogram showed a sinus heart rate of 69 beats per minute. The heart ultrasound showed no significant abnormalities in cardiac structure and blood flow. The electromyogram demonstrated normal right anterior tibial nerve insertion potential, incomplete light and strong contraction, the right dorsal inter bone muscle and bilateral medial femoral myoneurogenic injury, and myogenic damage. Multifocal lesions were detected on MRI by a decreased signal on the T1 sequence, increased signal intensity on the T2/FLAIR, and diffusion-weighted imaging (DWI) sequence showed a of the bilateral basal, centrum ovale, frontal lobe, and parietal lobe, and increased signal on the T2 sequence showed a of ganglia cervical cord (Figure 1).

**FIGURE 2:**
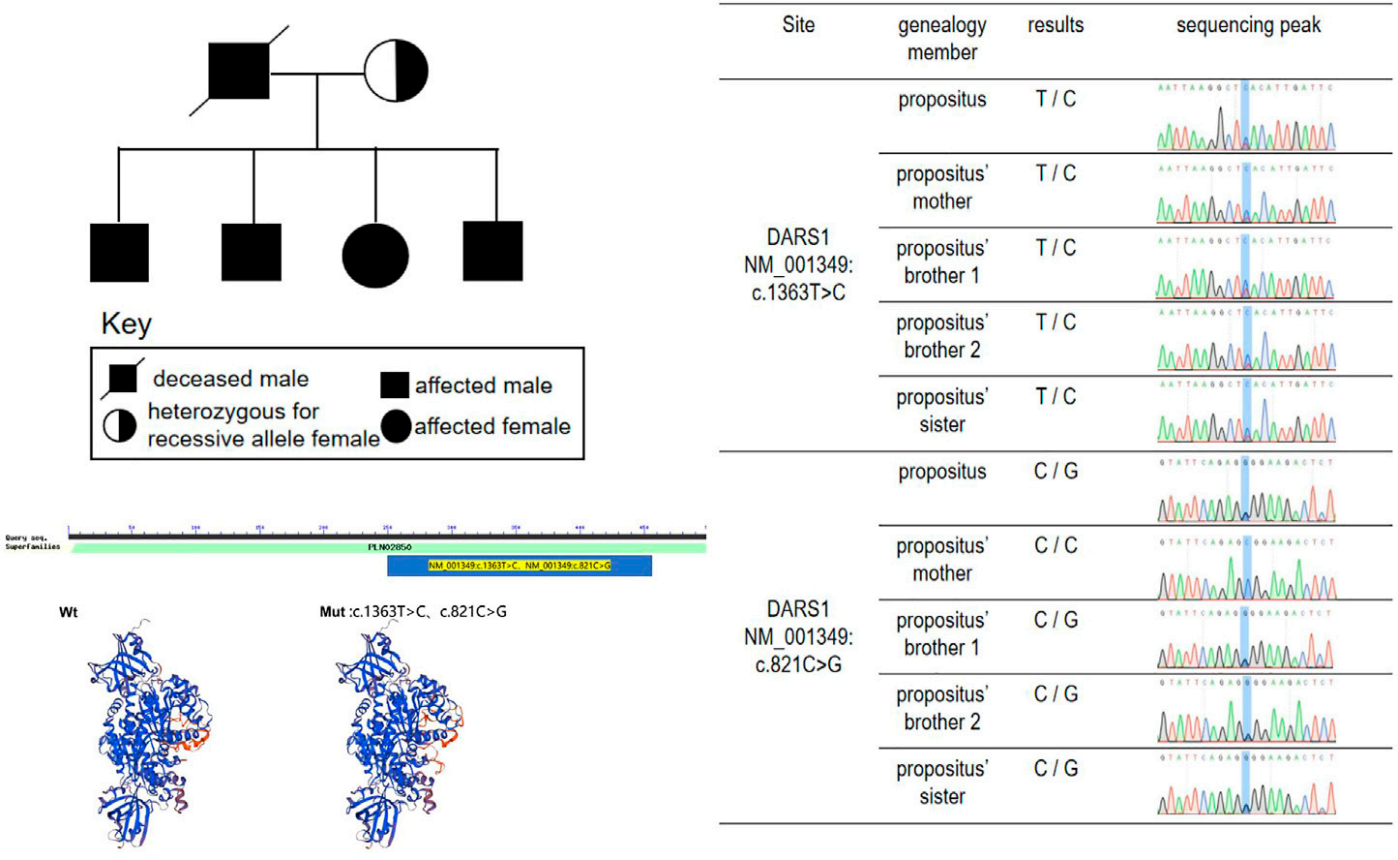
The genetic mutation and genetic lineage map.

Whole-exome sequencing analysis was performed using whole-exome capture with NimbleGen2.0, detecting the distribution and concentration of qPCR amplified libraries by AgilentBioanalyzer2100 and Hiseq2500 sequencing. Variant annotation was performed using the PolyPhen-2.2.2 software, ANNOVAR software, HGMD, dbSNP, and 1,000 genome database. Mutations in DARS1 detected were, c.1363T > C (p.Y455H) (of maternal origin) and c.821C > G (p.A274G). Moreover, his elder brothers and sister’s mutations in DARS1 detected were c.1363T > C (p.Y455H) and c.821C > G (p.A274G). And his mother’s mutations in DARS1 were detected, namely, c.1363T > C (p.Y455H). The two elder brothers and sister of the proband developed weakness of lower limbs and walking unstable, when they were teenagers. The mother of proband has no obvious clinical symptoms at present, but it couldn not rule out the possibility she has no clinical symptoms in the future. (Figure 2). The mutations in DARS1 of the proband and his sibings were c.1363T > C (p.Y455H) (of maternal origin) and c.821C > G (p.A274G) (of paternal origin), and his father died when he was 12 and his gene could not be detected. We speculated that the cause of his father’s death was gene mutation (Figure 2; Table 1). The mutations are termed as “pathogenic” and “likely pathogenic” according to the American College of Medical Genetics and Genomics (ACMG) guidelines, and a diagnosis of HBSL was made. The c.1363T > C (p.Y455H) gene was obtained from his mother.

**TABLE 1:** DARS Mutations associated of the Patient.

Genomic Position (hg19)	Nucleotide Alterations	Exon	Protein Alteration	Inheritance
chr2:136	NM_001349	15	p.Y455H	Maternal
6,68,760	c.1363T > C			
chr2:136	NM_001349	10	p.A274G	paternal
6,78,161	c.821C > G			

Since HBSL is characterized by hypomyelination, the treatment is essential to promote myelin repair. The patient was prescribed vitamin B1 (Yangzhou Eddie Pharmaceutical Co., Ltd.) 10 mg orally three times a day, methylcobalamin tablets (Wei Material, China Pharmaceutical Co., Ltd.) 0.5 mg three times a day, and cytophosphate choline sodium tablets (Sichuan Zitonggong Pharmaceutical Co., Ltd.) 0.2 g three times a day for a year. On a follow-up visit after 1 year, the patient could lift heavy weight and ambulate with the support of crutches.

## 3 Discussion

The characteristic manifestation of HBSL is that severe spasms occur in the first year of life and motor paraparesis occur, resulting in difficulty in walking independently. HBSL symptoms also include hypoevolutism in motor development, nystagmus, and cognitive developmental delays. HBSL is a rare hereditary disease, and only a few cases of infants or children with HBSL have been reported. Since this patient failed to receive medical aid in early childhood, he was diagnosed with HBSL in adulthood. At present, he is the oldest patient reported on a global scale, has a 43-years course of the disease, involving mobility of lower limbs, and upper limbs with normal cognitive function. This further reinforces the fact that the main clinical symptom of HBSL is limb paralysis and has little effect on cognitive function. The main clinical symptoms of classic early-onset HBSL are delayed motor development, progressive lower limb spasm, inability to walk, and normal cognitive function. Patients with the late-onset disease are mainly teenagers, with symptoms of dyskinesia and lower limb spasm. Before the onset of the disease, their movements, and cognitive functions are normal. MRI shows symmetric high signal intensity in T2W images in bilateral periventricular white matter. Subcortical white matter, corpus callosum, or internal capsule is not involved (Zhang et al., 2018). The disease in our patient started in early childhood, which is also evident by the radiological features matching the description of the classic early-onset HBSL.

**FIGURE 3:**
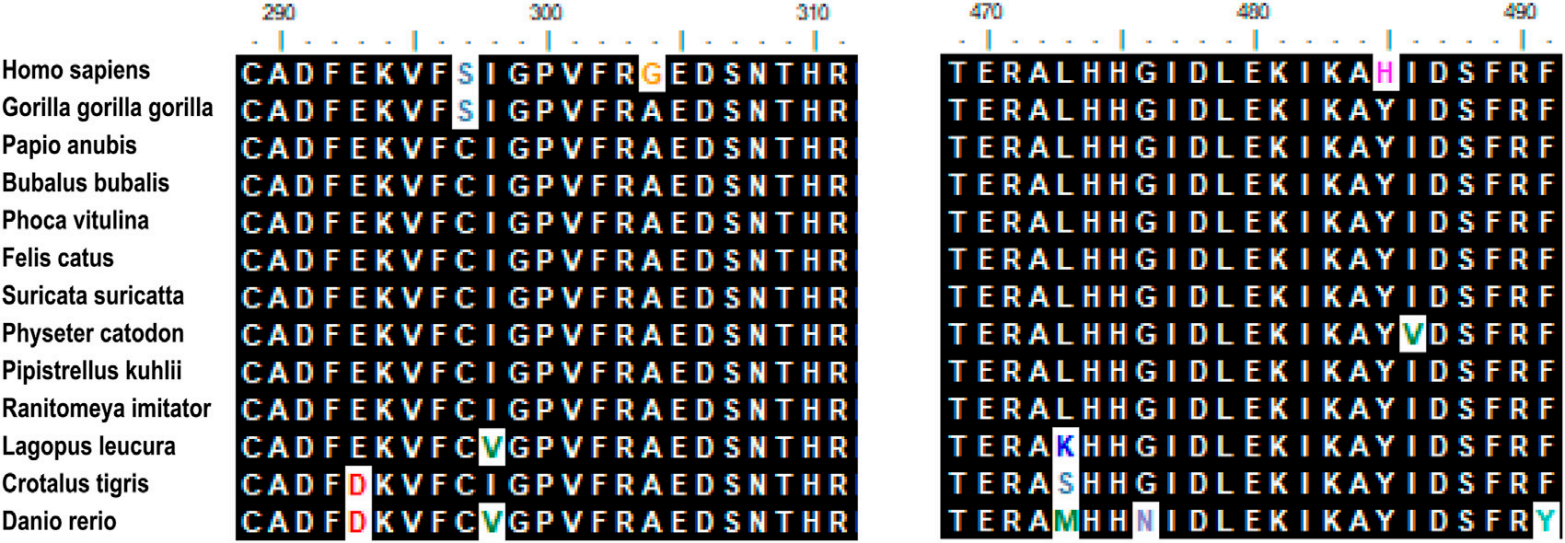
Sequence alignment of the DARS in different species. Comparison of the amino acid sequence show its high conversation among species. Amino acids that differ from the sequence of the insert in humans are indicated in different colors. Mutation site (N304 and N485) are marked in yellow and purple.

Hypomyelination with brain stem and spinal cord involvement and leg spasticity (HBSL) is a leukodystrophy caused by missense mutations of the aspartyl-tRNA synthetase-encoding gene DARS1. Homozygous as well as compound heterozygous point mutations of the DARS gene that confer non-synonymous amino-acid substitutions to highly conserved residues in the catalytic domain cause the white matter disorder Hypomyelination with Brain stem and Spinal cord involvement and Leg spasticity (HBSL) (Ryan et al., 2013; Nicole et al., 2015). The mutant gene associated with HBSL is DARS, located on the 2q21.3 chromosome. The DARS gene encodes the cytoplasmic aspartyl-tRNA synthetase (Kim et al., 2013), which functions as a dimer and consists of an N-terminal anticodon recognition domain and a C-terminal catalytic domain. The mutations in tRNA synthetases can cause a broad range of neurologic disorders. It’s the first time that HBLS-related mutant genes were reported by Ryan et al. They performed an investigation of DARS expression in humans and mice by using a curated set of publicly available data found that DARS is a core component of the translational machinery, and it is diffusely localized in cytoplasm and broadly expressed in the central nervous system. These data are consistent with HBSL’s clinical presentation. (Ryan et al., 2013). The encoded protein is highly expressed in brain tissue, especially the cerebellum, brain stem, thalamencephalon, hippocampus, basal ganglia, and spinal cord (Dominik et al., 2017). A study on a biopsied sample of the cerebellum of a patient with HBSL has demonstrated a very high expression of the DARS gene (Dominik et al., 2018). The study of the expression of DARS in the central nervous system is crucial for the treatment of HBSL using targeted therapies. One study analyzed endogenous DARS expression on the mRNA and protein level brain and in human stem cell-derived neurons, oligodendrocytes, and astrocytes (Dominik et al., 2018). Oligodendrocyte plays an important role in myelination; hence DARS gene mutation results in the demyelination of white matter by decreasing the expression of endogenous DARS in oligodendrocytes. Gene mutations were also detected in DARS1 in this patient. The full length of DASR1 coding sequence is 1506 bp, and the protein sequence length is 501 amino acids. We found the patient’s mutation position occurs at the 821st base position of the coding sequence (C-G), resulting in the amino acid changing from alanine (Ala) to glycine (Gly), in addition, at the 1363st base position of the coding sequence (T-C), resulting in the amino acid tyrosine (Tyr) becoming histidine (His). The two point mutations result in amino acid changes (Table 1). Sequence alignment of the DARS in different species. Comparisons of the amino acid sequence show its high conservation among species(Figure 3).

Data available on the treatment of HBSL at present is lacking. Mecobalamin participates in the synthesis of the myelin, which is one of the important components of the myelin sheath. Mecobalamin can improve the activity of methionine synthetase and promote the synthesis of lipid lecithin, which is one of the main structures of the myelin sheath, thereby improving the formation of the myelin sheath, promoting Schwann’s cell metabolism, and repairing the damaged myelin sheath (Ma et al., 2010). Citicoline is a nucleic acid derivative that is enzymatically catalyzed to produce phosphatidylcholine, a part of the bilayer lipid membrane. Moreover, citicoline plays an important role in the synthesis of lecithin and improves nerve function by promoting the synthesis of lecithin. When the nerve cell membrane is damaged, exogenous citicoline is continuously supplemented to synthesize phosphatidylcholine, which participates in the repair of the nerve cell membrane (Jain-bo et al., 2010). Phospholipids, including phospholithin, cerebral phospholipid, and sphingolipids are one of the important components of the lipid layer and myelin sheath is composed of protein and lipid (Richard, 2002). Moreover, phosphatidylcholine and sphingolipid in the nerve cell membrane are degraded if choline is deficient, leading to cell apoptosis. Continuous supplementation of choline in exogenous cytohocholine catabolism can prevent nerve cell membrane damage and cholinergic neurodeath (Yen et al., 1999). In this case, after treatment with vitamin B1, methocobalamin, and cytophosphocholine sodium, an improvement in limb movement was observed. We conclude that mecobalamin and vitamin B1 could promote myelination and may be effective in HBSL. However, comprehensive clinical research is still needed to confirm it in the future.

## Data Availability

The original contributions presented in the study are included in the article/Supplementary Material, further inquiries can be directed to the corresponding authors.
